# Mapping reduced introgression loci to the X chromosome of the hybridizing field crickets, *Gryllus firmus* and *G*. *pennsylvanicus*

**DOI:** 10.1371/journal.pone.0208498

**Published:** 2018-12-19

**Authors:** D. Patrick Gainey, Jeremiah Y. Kim, Luana S. Maroja

**Affiliations:** Department of Biology, Williams College, Williamstown, Massachusetts, United States of America; National Cheng Kung University, TAIWAN

## Abstract

The genomic architecture of barriers to gene exchange during the speciation process is poorly understood. The genomic islands model suggests that loci associated with barriers to gene exchange prevent introgression of nearby genomic regions via linkage disequilibrium. But few analyses of the actual genomic location of non-introgressing loci in closely related species exist. In a previous study Maroja *et al*. showed that in the hybridizing field crickets, *Gryllus firmus* and *G*. *pennsylvanicus*, 50 non-introgressing loci are localized on two autosomal regions and the X chromosome, but they were not able to map the loci along the X chromosome because they used a male informative cross. Here, we localize the introgressing and non-introgressing loci on the X chromosome, and reveal that all X-linked non-introgressing loci are restricted to a 50-cM region with 10 of these loci mapped to a single location. We discuss the implications of this finding to speciation.

## Introduction

Although the basic characteristics of barriers to gene exchange are relatively well understood, the underlying genomic architecture of such barriers is not. Investigation of the localization of alleles or chromosomal rearrangements associated with reduced gene flow can give us insight into the genomic architecture of barriers to gene exchange and help us better understand the speciation process. For instance, are highly differentiated loci spread evenly across the genome, or are they concentrated in few areas following an “islands of speciation” model? Are there certain genes or chromosomes that often show signatures of restricted introgression between hybridizing species? What is the nature of loci unable to cross species barriers? The field crickets *Gryllus firmus* and *Gryllus pennsylvanicus* are an excellent system in which to study the genetic architecture of introgressing and non-introgressing loci. These crickets form an extensive, well-characterized hybrid zone with multiple barriers to gene exchange including many prezygotic barriers and a one-way reproductive incompatibility, likely a post-mating, prezygotic barrier [1, 2, 3, 4, 5]. The pair of species diverged recently (~200,000 years) and 110 loci have been characterized in two locations in the hybrid zone [5, 6].

Larson et al. [5, 6] showed that most of the loci are able to freely flow between species, but 50 (out of 110) display sharp genomic and geographical clines in both the Pennsylvania and Connecticut hybrid zones. Maroja *et al*. [7] determined that of these non-introgressing loci, 9 and 2 loci were mapped to small regions of the autosomal linkage groups LG14 and LG2, respectively. The rest of the non-introgressing loci (n = 39 out of 50) were found to be X-linked [7]. However, as the backcross used by Maroja *et al*. [7] was male-informative (an F1 male backcrossed to a *G*. *pennsylvanicus* female), the X-linked loci could not be ordered along the X chromosome. Here we built a new genetic map focusing only on the X-linked loci using a female informative cross (F1 female backcrossed to a *G*. *pennsylvanicus* male).

## Materials and methods

All steps in the data acquisition and analyses closely followed the protocol from Maroja *et al*. [7]. We collected pure species crickets from Ithaca, NY (42°24’, -76°31’) (*Gryllus pennsylvanicus*, *GP*) and Guilford, CT (41°15’, -72°42’) (*Gryllus firmus*, *GF*) in August 2014. These crickets are not an endangered or protected species and thus do not required collection permits. Virgin *GP* females were crossed to *GF* males and resulting F1 female hybrids were mated to captive hatched *GP* males. Eggs were incubated at 4°C for 2 months prior to hatching at room temperature. We preserved two-week-old backcross offspring from the single, most fertile female in 100% ethanol in -20°C.

### DNA extraction and library preparation

We used Agencourt DNAdvance Genomic DNA Isolation Kit (Beckman Coulter, Inc.) to extract DNA from 163 backcross nymphs and their parents. A total of four offspring failed to generate enough reads for successful analyses and were eliminated.

We sequenced individuals in three Illumina Miseq lanes (Cornell Life Sciences Core Laboratory Center for first batch of individuals, Genewiz for second and third batch). For each individual, we first performed 5 PCR reactions with 24 primer pairs in each using the Qiagen Multiplex PCR Kit (Qiagen) to amplify a total of 120 SNP markers, the 165 individuals were divided into two Illumina lanes. To each individual we added Illumina barcodes (N501-520/S701-729) via PCR. All barcoded individuals from each batch (lane) were combined in a single mix and cleaned up using Agencourt Ampure XL beads (Beckman Coulter, Inc.). The third Illumina lane was used to get data for loci that failed to generate enough reads in the previous runs (23 loci).

### Analyses of sequences

We analyzed sequences using Geneious R9 (Biomatters, https://www.geneious.com). All reads were mapped to a library containing reference sequences of all amplified loci. Consensus sequences were generated for each amplified locus/individual using a threshold of 75%. All consensus sequences were then grouped into a single list, which was then mapped to the reference sequence library with medium sensitivity and up to five iterations. Of the 120 sequenced loci (58 X-linked loci), we were able to generate usable data for nearly all analyzed individuals at 91 loci (44 X-linked loci), the remaining loci did not generate enough reads in many individuals and were thus eliminated.

### Construction of linkage map

We analyzed genotype data using JoinMap 4.1 [8]. Linkage groups were determined using logarithm of odds (LOD) calculations with a threshold of 4.0. Multiple regression mappings were done on X chromosome linkage group, yielding similar maps. A total of three remaining X-linked loci did not follow Mendelian expectations and were excluded from these final maps (Chi-square, P<0.01). Data used for mapping and unformatted SNPs can be found in Dryad doi: 10.5061/dryad.h0g0kf0.

## Results and discussion

**Fig 1:**
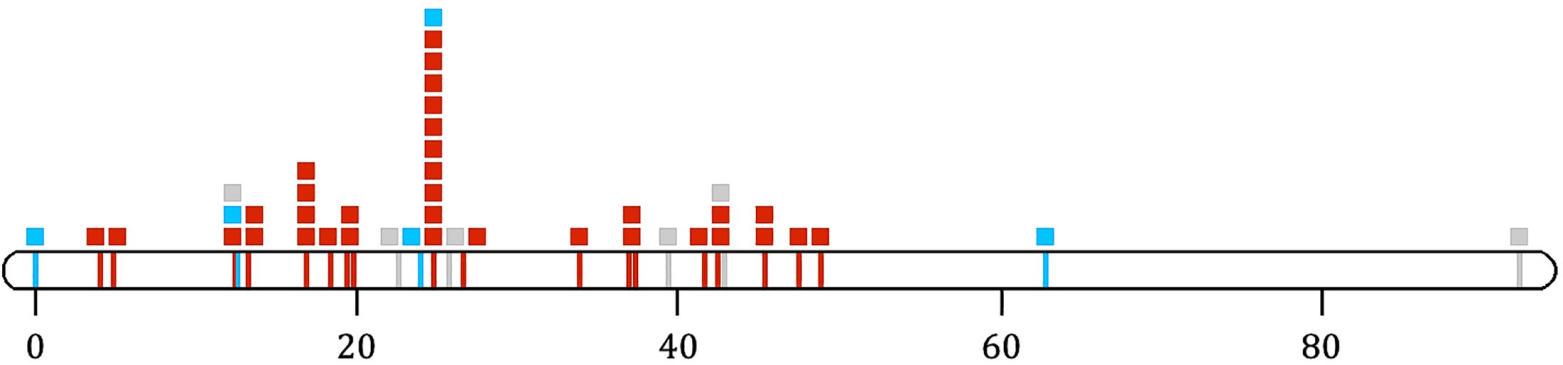
Map of introgressing, non-introgressing loci on X chromosome. Red boxes, ticks = non-introgressing loci, blue boxes, ticks = introgressing loci, grey boxes, ticks = not analyzed by Larson *et al*. (2013, 2014). Height of box stacks indicates number of SNPs mapped to a given location. Distances measured in cM from leftmost locus.

Our map revealed a single linkage group of 92cM representing the 44 X linked loci. This single X linkage group (LG) merged the two separate LG representing the X chromosome in Maroja *et al*. [7] (S1 Table). The X chromosome was shorter than that reported in Maroja *et al*. [7], in which the largest X LG was 106cM. The underestimated distance between loci is likely due to the reduced number of loci (we did not include a new ddRAD library) in our analyses leading to a reduced number of 3-point crosses. Of the 41 previously-analyzed X linked loci, 33 were non-introgressing. All of these 33 non-introgressing loci were located within a 50-cM region but 10 of them mapped to a single location (Fig 1). A similar clustering of non-introgressing loci was previously observed on autosome LG14 [7]. Interestingly, some introgressing loci mapped to areas of the X chromosome that were highly enriched for non-introgressing loci. This was an unexpected result because loci in the same position are expected to be under high linkage disequilibrium and thus experience similar rates of introgression or lack thereof.

The observed clustering of non-introgressing loci into short regions of LG14 and the X chromosome follows the genomic pattern predicted by the genomic islands of speciation model. This model states that alleles that act as barriers to gene exchange will, via linkage disequilibrium, ensure that genomic regions in close proximity also fail to be exchanged between species. Eventually, mutations will accumulate within these regions and the regions’ boundaries will expand, further reducing gene flow [9] to eventually encompass the entire genome. Critics of this model argue that observed examples of genomic islands could instead be caused by a combination of ancestral variation and selection in areas of the genome with restricted recombination [10, 11], leading to the observation of “islands of differentiation” even in the complete absence of introgression. Either of these explanations could account for the island pattern of non-introgressing loci observed in *Gryllus* but would imply very different speciation histories. In the first case introgression would have been important in homogenizing genomes and non-introgressing areas would be free to accumulate mutations. In the second case hybridization (while certainly happening) did not play a role in introgression and homogenization, instead selection led to the differentiation of genomes that were initially similar due to common ancestry.

In Maroja *et al*. [7], the *Gryllus* X chromosome was found to be enriched both for highly differentiated loci and for non-introgressing loci. This might be due to several factors. First, the SNPs analyzed across the hybrid zone and mapped in this study were derived from a male accessory gland RNAseq library, and the chosen SNPs were those with a relatively strong differentiation between allopatric populations of the two species, near fixation [5, 6, 12]. Thus, if male-specific genes expressed in the accessory gland are overrepresented on the X chromosome, we would likely see a large number of SNPs localizing to the X. Furthermore, the larger differentiation of the X-linked loci could be due to its smaller effective population size (3/4Ne), which is predicted to increase genetic drift and thus accelerate population differentiation [13]. Additionally, male-hemizygous recessive alleles on the X are subjected to more intense selection [14], which may again lead to higher differentiation if differential selection exists between populations.

Similar to our observation that there is a specific region with reduced introgression on the *Gryllus* X chromosome, the human X chromosome displays large stretches with greatly reduced levels of Neanderthal ancestry [15]. A similar region of reduced introgression is also seen in the X chromosome of house mice [14]. In these two mammalian species, it has been shown that the X chromosome is enriched for loci associated with decreased fertility or male hybrid sterility, suggesting that the X chromosome might often harbor genes responsible for barriers to gene flow or species differentiation [15, 16]. For example, mammalian spermatogenesis is a process known to have elements under positive selection, and in mice the X chromosome is significantly enriched for genes uniquely expressed in spermatogonia, the pre-meiotic germ cells of the testis from which sperm arise [17, 18]. As is the case in mammals, the orthopteran X chromosome seems to be inactive in the male meiotic germline [19, 20, 21, 22]; it is thus possible that the Orthoptera X is not enriched for spermatogenesis genes. However, it is still possible that X-linked accessory gland genes are expressed and, in other *Drosophila*, accessory gland proteins have been shown to be essential in fertilization, fecundity and female postmating behavior (e.g. [23, 24]). Furthermore it is possible that X-linked gene products accumulate prior to meiosis thus, even if the meiotic X is inactive, its products could still play a role in sperm production (as might be the case with mammals). Collectively, these factors indicate that contributions from both the male-hemizygosity of the X chromosome and the genomic trend for localization of sterility factors to the X may both contribute to the observed enrichment of non-introgressing loci to the X chromosome.

It must be noted that the 50-cM region in which non-introgressing loci are spread on the X chromosome is far larger than the autosomal region with non-introgressing loci (only 7-cM in LG14, [7]). This would be consistent with a larger number of X-linked loci acting as barriers to gene exchange compared to autosomal loci. Alternatively, the large size of this highly differentiated region may simply be due to the higher level of linkage disequilibrium on the X chromosome, which has been associated with the lack of recombination and increased selection pressure of the X chromosome in hemizygous males [25]. Thus, even if only a few loci were unable to cross species barriers, many other loci that do not directly affect fitness of the other species could hitchhike with them due to the lower levels of recombination, a phenomenon that would be less prevalent on autosomes.

It is also possible that one or more chromosomal inversions are behind the clustering of many loci in the X chromosome. This would provide an alternative explanation for the shorter length of our X chromosome map in relation to that in Maroja *et al*. [7]; the previous map was a result of recombination between the two *G*. *pennsylvanicus* chromosomes while our current map is a result of recombination between chromosomes from different species, potentially involving inversions. Chromosomal inversions are known to play a role in population and morph differentiation, such as in "social" chromosome of fire ants [26] and the ruff mating system [27]. Inversions also play a role in speciation and ecotypification, such as in *Drosophila pseudoobscura* and *D*. *persimilis* [28], in *Anopheles gambiae* [29], and in *Gadus morhua* [30]. Of particular relevance, a recent study showed that the number of inversion differences between species of passerine birds is associated with range overlap and that in passerines inversions are more likely to be fixed on the Z chromosome than on the average autosome [31]. It is thus possible that one or more inversions on the *G*. *firmus*/*G*. *pennsylvanicus* X chromosome may have played a role in the reduction of gene exchange in the X chromosome and might potentially have contributed to the further development of barriers to gene exchange between the two species.

## Supporting information

S1 Table Exact distances of all mapped X-linked loci. Distance is measured in cM from leftmost locus (seq1412) on the X chromosome. Data can be found in Dryad: doi: 10.5061/dryad.h0g0kf0.
(XLSX)
